# Cancer Environment Immunotherapy: targeting TGF-β finds its way towards tissue healing and vasculature remodeling

**DOI:** 10.1038/s41392-021-00472-z

**Published:** 2021-01-29

**Authors:** Ke Jin, Fangfang Zhou, Long Zhang

**Affiliations:** 1 Laboratory of Human Diseases and Immunotherapies, West China Hospital, Sichuan University, Chengdu, China; 2 Institutes of Biology and Medical Science, Soochow University, Suzhou, China; 3 MOE Laboratory of Biosystems Homeostasis and Protection and Innovation Center for Cell Signaling Network, Life Sciences Institute, Zhejiang University, Hangzhou, China

**Keywords:** Immunotherapy, Cancer therapy

Recently, Prof. Ming O. Li and colleagues published two back-to-back studies in *Nature*, demonstrating that blocking transforming growth factor β (TGF-β) signaling in CD4^+^ T cells leads to tissue healing and remodeling of the blood vasculature, causing cancer cell hypoxia and death in distant avascular regions.^1^ Furthermore, they also provide direct evidence validating that the above strategy does restrain cancer progression in a mouse model of breast cancer resistant to immune-checkpoint or anti-VEGF therapies.^2^

Cancer immunotherapy, represented by immune-checkpoint blockade (ICB) and chimeric antigen receptor T (CAR-T) cell therapy, has brought revolutionary changes to the field of cancer treatment. A portion of patients with advanced cancers who did not respond to traditional chemotherapy, radiotherapy and targeted therapy obtained satisfactory prognosis after receiving anti-PD-1, PD-L1, or CTLA-4 antibodies.^3^ However, a large number of clinical trials have shown that, except for specific cancer types, such as non-small cell lung cancer and melanoma, most of the cancer patients do not respond well to ICB.^3^ Therefore, researchers are still prompted to find new cancer immunotherapies or ways to improve the efficacy and expand the indications of the current therapies.

**Fig. 1:**
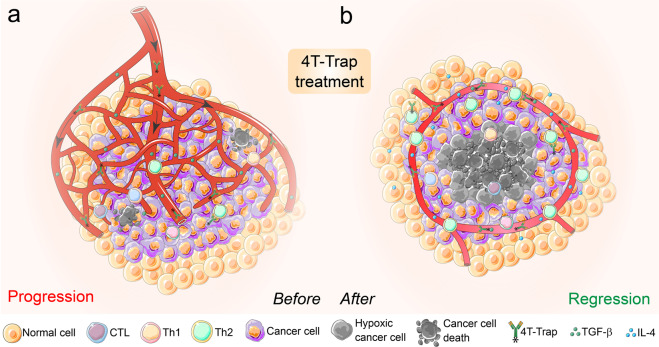
Blockage of TGF-β signaling in CD4^+^ T cells by 4T-Trap reprograms tumor vasculature and halts cancer progression. **a** In a well-developed solid tumor (e.g., breast cancer), the irregular and abundant vasculature in the local “hotbed” could fully support tumor growth and compromise the outcome of cancer therapies. **b** Blockade of TGF-β signaling in CD4^+^ T cells with 4T-Trap results in enhanced Th2 cell differentiation that promotes vasculature remodeling and tumor tissue healing with cancer cell hypoxia and death in avascular regions. 4T-Trap probably represents a novel modality of “cancer environment immunotherapy”

In the present two studies, Prof. Ming O. Li et al. provide an alternative and “indirect” therapeutic strategy targeting the “hotbeds” of tumors, the tumor microenvironment (TME), instead of directly boosting the cytotoxic or killing capacity of tumor-specific T cells.^1,2^ Previously, they found that blocking TGF-β signaling in T cells inhibited tumor progression,^4^ and now they aim to figure out which type of T cell actually mediates this antitumor immune response triggered by TGF-β blockade. By specifically depleting TGF-β receptor 2 (TGFBR2) in CD4^+^ or CD8^+^ T cells from MMTV-PyMT transgenic mice that will develop spontaneous breast cancer, they surprisingly discovered that it was CD4^+^ T cells, not CD8^+^ T cells, that halted cancer progression when their internal TGF-β signaling was blocked.^1^ Further experiments demonstrated that tumor tissues from *Thpok-cre;Tgfbr2*^*fl/fl*^ PyMT mice that had deficient TGF-β signaling in CD4^+^ T cells showed less extravascular deposition of fibrinogen and irregularly shaped microvasculature with more hypoxia-associated cancer cell death compared to that from *Tgfbr2*^*fl/fl*^ PyMT mice.^1^ Interestingly, the hypoxic areas exhibited a circular pattern inside the reorganized vessels and outside the regions of cancer cell death in the tumors from *Thpok-cre;Tgfbr2*^*fl/fl*^ PyMT mice, a process that a bit looked like “tumor tissue healing” (Fig. 1).^1^ More interestingly, this robust antitumor immunity triggered by TGF-β signaling blockage in CD4^+^ T cells was dependent on the characteristic cytokine of type 2 immunity IL-4 but not on the traditional tumor-killing cytokine IFN-γ (Fig. 1).^1^

Overall, this basic research has three highlights and novel implications. First, it provides one more strong evidence that CD4^+^ T cells are not just the supporting roles in antitumor immunity compared to CD8^+^ T cells, and even outperform CD8^+^ T cells under certain circumstances.^5^ Second, it offers direct evidence that type 2 immune response suppresses tumor development in an unexpected manner, underlining its prominent role in restraining angiogenesis and tumor progression in subsets of cancer patients.^1^ Lastly, it provides a new strategy for targeting TGF-β signaling in the TME. TGF-β is a well-known pleiotropic cytokine regulating numerous pathophysiological processes including carcinogenesis and wound healing.^1^ Previous targeted therapies targeting TGF-β did not achieve much success, partly due to the severe side effects caused by TGF-β signaling blockage.^2^ In the second translational research by Prof. Ming O. Li et al., they fully elucidated how to pharmacologically block TGF-β signaling in CD4^+^ T cells and elicit reorganization of tumor vasculature and cancer cell death following the above findings.^2^

First, they constructed a bispecific receptor decoy by attaching the TGF-β-neutralizing TGFBR2 extracellular domain to ibalizumab, a non-immunosuppressive CD4 antibody used as an antiretroviral drug, and named it CD4 TGF-β Trap (4T-Trap).^2^ 4T-Trap was proven to preserve efficient CD4 binding and potent TGF-β-signaling inhibition in vitro and in vivo.^2^ In a mouse model of breast cancer resistant to immune-checkpoint or anti-VEGF therapies, 4T-Trap caused significant inhibition of tumor growth and vasculature branching following 5–6 weeks’ treatment, triggering catastrophic cancer cell death in hypoxic areas away from the vasculature (Fig. 1), whereas the irregular tumor growth and angiogenesis were increased in all control groups.^2^ Mechanistically, 4T-Trap treatment led to increased proportions of effector-memory CD4^+^ T cells in tumor-draining lymph nodes and enhanced differentiation of IFN-γ-producing Th1 and IL-4-producing Th2 cells; however, only neutralization of IL-4 fully reversed the tumor-repression phenotype, further highlighting the important function of type 2 immune responses in suppressing tumor development.^2^

It is worth noting that 4T-Trap also upregulated vascular endothelial growth factor A (VEGFA) expression in hypoxic areas of tumors from tumor-bearing mice, which might be a result of cellular adaptation to resolve ischaemia and counteract the tumor inhibition effects of 4T-Trap.^2^ Therefore, the authors designed a VEGF receptor decoy containing fragments of VEGF receptors fused to a mouse IgG2a Fc and called it VEGF-Trap, which could diminish tumor vessel density, but did not affect vessel patterning.^2^ Further experiments validated that coadministration of VEGF-Trap with 4T-Trap synergistically resulted in expanded regions of cancer cell death to the outer boundary of the hypoxic areas, indicating that 4T-Trap can be combined with VEGF inhibitors to further restrain tumor-vasculature-mediated cancer progression.^2^

The immune system protects us not only by directly eradicating pathogens and transformed cells, but also via promoting tissue repair and inflammation resolution, and now the latter mechanism could also be exploited to fight against cancer.^1^ Taken together, these two interconnected studies probably show a new targeted antitumor immunity landscape towards the next-generation of cancer immunotherapy, which takes advantage of TGF-β signaling blockade in CD4^+^ T cells, triggers tissue healing around tumors, reshapes tumor vasculature, cuts off nutrient supply, and ultimately eliminates cancer growth (Fig. 1).^1,2^ This “cancer environment immunotherapy” may become a new type of cancer immunotherapy, or at least serve as an important supplement to the existing cancer therapies.
